# A Study on the Factors Affecting the Prescription of Injection Medicines in Iran: A Policy Making Approach

**DOI:** 10.5539/gjhs.v7n3p291

**Published:** 2015-01-14

**Authors:** Mohammad Meskarpour-Amiri, Nooredin Dopeykar, Parisa Mehdizadeh, Ali Ayoubian, Zahra Motaghed

**Affiliations:** 1 Health Economics Department, Health Management Research Center, Baqiyatallah University of Medical Sciences, Tehran, Iran; 2 Management and Economics School, Tarbiat Modares University, Tehran, Iran; 3 Research Center for Modeling in Health, Institute for Futures Studies in Health, Kerman University of Medical Sciences, Kerman, Iran; 4 Health Research Center, Baqiyatallah University of Medical Sciences, Tehran, Iran; 5 Student Research Center, Baqiyatallah University of Medical Sciences, Tehran, Iran

**Keywords:** prescription, injection medicines, health policy

## Abstract

**Background and Aim:**

Inappropriate prescribing injection medicines can reduce the quality of medical care, patient safety, and leads to a waste of resources. Sufficient evidence is not available in developing countries to persuade policy-makers to promote rational drug prescription.

The objective of this study is to assess some factors affecting the prescription of the injection medicines in Iran.

**Methods:**

In this descriptive-analytic study, the data of 91,994,667 selected prescription letters were collected by the Ministry of the Health and Medical Education (MOHME) throughout the country at the year 2011 which were analyzed through a logarithmic regression model.

**Results:**

Results of the study show that the percentage of the prescription letters containing injection items varied from 27 percent (in Yazd) to 57 percent (in Ilam). Also the impact of price on the prescription of the injection medicines was not significant (P=0.55). But the impact of the prescription of antibiotics and corticosteroid on injections were significant (P>0.05) and equal 0.44 and 0.65 respectively.

**Conclusion:**

Increasing price of injection medicines as a policy towards reducing consumptions cannot be a successful policy. But reducing the use of antibiotics and corticosteroids can be a more effective policy to reduce the use of injection medicines.

## 1. Background

The optimization of the medication consumption patterns and stepping towards a society with appropriate, risk-free, effective, and quality consumption is the core content of the WHO recommendations worldwide (Jackson, Mangoni, & Batty, 2004). However nowadays, overuse of injection medicines is a health dilemma in developing countries. It is estimated by the WHO that about 16 billion injections are undertaken in developing countries annually (World Health Organization, 2004). According to a study that was conducted by the WHO, the average number of medicines prescribed in letters have been 2.1 in Nepal, 3.8 in Nigeria, 2.2 in Tanzania, 1.8 in Malaysia, 3.3 in India and 1.5 in Yemen. Also the percentage of the prescriptions containing injection medicines have been 5 in Nepal, 37 in Nigeria, 29 in Tanzania, 19 in Malaysia, 17 in India, and 25 in Yemen (World Health Organization, 1993). According to a study on the comparison of the injection medicines in the northern, central, eastern, western and southern areas of Tehran in 1999, the average prescription items for each prescription letter was equivalent to 3.6 which %39 of it consisted of injection medicines (Dinarvand & Nikzad, 2000). A study by Mosleh et al., that was conducted in 2004, for assessing the medication prescription status according to the WHO guideline on the standards of drug prescriptions, revealed that the average prescribed items were equivalent to 2.85 and at least one injection item was among 28.96% of them (Mosleh, Darbooy, Khoshnevis Ansari, & Mohammadi, 2008).

So far, there is no standard for the prescription quality indicators worldwide. Differences in the proportion of injections are seen in different countries (Hutin, Hauri, & Armstrong, 2003; Rasool, Fahmy, Abu-Gharbieh, & Ali, 2010). A physician’s prescription pattern is a result of a series of complex factors, including internal characteristics and motivations as well as external factors like social environment and patient requirements. Majority of the studies reveal that in addition to clinical considerations there are a variety of factors that may influence the decision on the prescription of injection medicines (Kumari et al., 2008). Based on the previous studies, patients’ characteristics including age, social and economic status, beliefs, and insurance status have considerable influences on prescription demand. Moreover, physicians’ characteristics including gender, age, time elapsed since graduation, and the duration of the practical education have significant effects on the prescription behavior. But features of medications such as medication categories and price have not yet been studied (Bharathiraja, Sridharan, Chelliah, Suresh, & Senguttuvan, 2005; Choi, Park, Lee, & Kwon, 2012).

Irrational prescription of medicines is one of the common problems of medical treatments in developing countries. As the injection is a process that requires supervision by skilled health care providers, its number is regarded very important. The excessive and unnecessary use of injection medications is not only a waste of medical costs, medical staff, time and medical equipment but also increases the risk of infection by viruses, like hepatitis C and AIDS (Beaney & Black, 2011; Kane, Lloyd, Zaffran, Simonsen, & Kane, 1999).

The Islamic Republic of Iran with an average annual medication consumption growth rate of 11.5% in comparison with the average growth rate of 7% among developing countries and 9% worldwide is one of the countries with the highest consumption rate of medical products (Abolhalaj, Bastani, Ramezanian, & Tamizkar, 2013). Furthermore, unleashed growth and consumption causes less supervision on the overuse of injection medicines and antibiotics. In this situation sufficient evidence is not available in developing countries to persuade policy-makers to promote rational drug prescription (Laing, Hogerzeil, & Ross-Degnan, 2001). Knowing the factors affecting the use of injection medicines can play a significant role in the policy making process in order to control the consumption and prescription of medicines. The objective of this study is to assess some factors that influence the prescription of injection medicines in Iran.

## 2. Materials and Methods

This research is a descriptive-analytic study that has assessed some factors that influence the prescription of injection medicines in Iran. The study used the data collected on 91.994.667 selected prescription letters throughout the country by the Ministry of Health and Medical Education (MOHME) published in 2011. The data had been separately collected from 36 cities or provinces with universities of medical sciences. The main variables in this study have been: the total number of prescription letters containing injection medicines, the total number of prescription letters containing antibiotics, the total number of prescription letters containing corticosteroids and the average price for each prescription letter.

Surveying the literature showed that multiple factors can influence the prescription of medicines and their injection form, but among them the prescription pattern and price of medicine are more important (Choi, Park, Lee, & Kwon, 2012; Scott, Mannion, Marshall, & Davies, 2003; Spurling et al., 2010). Different groups of medicines were prescribed according to the prescription pattern. Among them antibiotics and corticosteroids are the two main groups of medicine with more injection forms (Choi, Park, Lee & Kwon, 2012). Also price of medicines can play a considerable role in prescription especially in developing countries with more out of pocket health payments (Spurling et al., 2010). So in the present study the effect of prescription patterns (antibiotics and corticosteroid) and price of medicines were examined on the use of injections.

The data were entered to Excel 2007 in order to analyze descriptive statistics. Also for studying the relationship between variables of the study, we used a logarithmic regression model. This method provides a statistical process to examine the relationships between the study variables. The regression model has been used in previous studies to examine the factors influencing prescriptions and the use of drugs and medicines (Goldman, Joyce, Lawless, Crown, & Willey, 2006; Ulibarri et al., 2011). The main reason for choosing this method in the present study was that this method helps us to predict and forecast how the value of dependent variable change when each of the independent variables are varied. The regression model was as below:


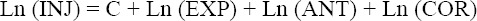


C= Constant Value

Ln= Natural logarithm

INJ = Prescriptions including injection medicines

EXP= Mean price of each prescription

ANT= Prescriptions including antibiotics

COR= Prescriptions including corticosteroid medicines

Eviews 5 software was used to estimate the regression model. Also the coefficients, P-Value, t-Statistic, and Standard Error were reported.

## 3. Results

Results of the study show that the average number of medicines in each prescription letter varied from 2.7 to 3.6 medicines per letter. 27 to 57 percent of all prescription letters contained injection medicines. Also the average price for each prescription letter varied from 30233 to 69986 RLs in different geographical regions. Table 1 shows the distribution of the prescription letters according to different geographical regions.

**Table 1:** Distribution of prescription letters in 36 cities or provinces with medical sciences universities at 2011

Provinces/Cites	Total number of prescription letters	Average price of each prescription letter (RLs)	% of prescription letters containing injection items	% of prescription letters containing corticosteroids	% of prescription letters containing antibiotics	Average number of medicines in each prescription letter
Ardebil	2513716	49843	43	24	53	3.27
Urmia	2724349	38593	53	35	50	3.29
Isfahan	11925581	52638	41	23	45	3.17
Ahvaz	11012397	43222	53	31	53	3.36
Ilam	399296	30223	57	34	56	3.22
Babol	526964	40606	44	26	51	3.24
Bojnord	456446	34618	40	18	52	3.10
Bushehr	514910	30766	28	19	44	2.74
Birjand	888549	40666	40	23	50	3.20
Tabriz	5578829	62218	44	28	49	3.38
Tehran	2582729	69986	33	17	41	2.89
Jahrom	84667	37148	27	16	39	2.80
Zabol	8,990	47207	50	32	61	3.64
Zanjan	1232461	35368	39	22	45	2.95
Sabzevar	303212	43693	34	15	43	3.20
Semnan	737033	38460	30	16	46	2.88
Shahrood	335333	33834	30	16	46	2.77
Shahrekord	1435022	41241	42	22	49	3.19
Shiraz	7606015	42266	38	20	47	3.10
Fasa	18802	37290	45	24	46	3.03
Qazvin	2322882	37725	52	30	49	3.52
Kashan	1325390	43365	47	29	51	3.49
Kurdistan	1091293	32113	43	25	51	2.97
Kerman	2326267	38053	29	17	46	3.01
Kermanshah	3134726	31869	43	24	52	3.20
Golestan	745060	44371	47	25	54	3.39
Gonabad	106355	37369	28	16	44	2.95
Gilan	4556977	51736	41	21	48	3.23
Lorestan	2160293	30780	50	29	54	3.36
Mazanadaran	5406897	46772	45	27	51	3.23
Markazi	197,831	35573	41	18	45	3.11
Mashahd	10069523	47693	37	19	46	3.14
Hormozgan	1188729	31721	35	20	52	3.00
Hamedan	2427129	53622	50	29	51	3.41
Yasuj	1883081	33561	42	23	52	3.01
Yazd	309933	52430	27	15	39	3.02

The estimation results for the regression equation showed that the impact of price on prescribing injection medicines was not significant (P=0.38). Also the impact of prescribing antibiotic and corticosteroid items on injection items was significant (P>0.05) and equal to 0.44 and 0.65 respectively. Also R^2^ statistics was 0.86 that means 86% of dependent variable changes can be justified with independent variables changes. Table-2 shows the estimation results for the regression model of the injection medicines prescription.

**Table 2:** The estimation results for the regression model of injection medicines prescription

Variables	Impact Coefficients	Standard Error	t-Statistic	P-Value
C	-0.69	1.16	-0.59	0.556
Ln (EXP)	0.063	0.07	0.89	0.380
Ln (ANT)	0.44	0.23	1.92	0.042
Ln (COR)	0.65	0.09	7.38	<0.001

R^2^=0.86.

## 4. Discussion

Inappropriate prescriptions of injection medicines can reduce the quality of medical care, patient safety, and leads to a waste of resources. Sufficient evidence is not available in developing countries to persuade policy-makers to promote rational drug prescription (Laing, Hogerzeil, & Ross-Degnan, 2001). The results of this study revealed that in Iran the average number of medicines in each prescription letter varies from 2.7 to 3.6 items for each prescription letter. The average number of medications per prescription in this study was markedly higher than the WHO’s recommended value of 1.3 to 2.0 (World Health Organization, 2004) The number of drugs prescription in this study is even higher than those of developing countries like India (3.3), Malaysia (1.8), Egypt (1.6), Yemen (1.5), Palestine (1.3), Indonesia (3.5) (World Health Organization, 1993). Although the pervious study by Soleymani et al) reported the average number of medications per prescription letter in Iran significantly decreased by 22.8% during 1998-2007 (4.25–3.28) (Soleymani, Valadkhani, & Dinarvand, 2009, But it seems that there is still a large gap between the existing situation and the WHO’s standards.

Besides, the percentage of the prescription letters containing injection items varies from 27 to 57 which was much higher than the WHO Standards and was much more than the WHO’s report for Malaysia (19%), India (17%), Yemen (25%), Nigeria (37%) and Tanzania (29%) (World Health Organization, 1993). Also it was much more than Tang’s study in China (20.02%) (Tang et al., 2013) and slightly more than Hogerzeil’s findings in Uganda, Sudan and Nigeria (36%–48%) (Hogerzeil et al., 2013). A review of the previous studies in Iran, confirms the difference in prescribing the injection medicines in different geographical areas (Dinarvand & Nikzad, 2000; Meymand, Sepehri, Farokhi, Beygim, & Motevali zadeh, 2008; Mosleh, Darbooy, Khoshnevis Ansari, & Mohammadi, 2008).

In a study on 4190 prescription letters in 2004, Mosleh et al estimated that the prescription letters containing injection items were equal to 28.9% (Mosleh, Darbooy, Khoshnevis Ansari, & Mohammadi, 2008) on average while Meymandi et al reported it equal to 36.8% in the same year in the city of Bam (Meymand, Sepehri, Farokhi, Beygim, & Motevali zadeh, 2008).

Conducted studies in the country show the descending trend for the prescription of injection medicines but in rural areas it’s remarkably lower than urban ones (Khaksari, Ahmadi Kohanali, Sepehri, Shafei, & Sadeghi, 2002; Sepehri & Meimandi, 2006). It can be concluded that in addition to the high percentage of injection items prescription in Iran, there are considerable differences based on the geographical regions. It seems that in such circumstances, in addition to trainings which aim to reduce the prescription and consumption of injection medicines, compiling a treatment protocol in order to reduce the geographical differences in drug prescription is necessary.

Results of estimation logarithmic equation in our study show that prescription of injection items is inelastic (not related) to price. It means that increasing or decreasing price of prescriptions has no significant effect on increasing or decreasing the prescription of injection medicines (P=0.55). The issue could be due to the nature of injection medicines as an essential good and the lack of attention to price of medicine at the time of prescription. The studies which were conducted in order to estimate the function of medicine demand in Iran, showed that the medication is an essential good for families in Iran and its price and income elasticity is lower than 1 (EbadifardAzar, Rezapoor, Rahbar, Shokouh, & Morteza, 2013; Ravangard, Jafari, & Motlagh, 2014). Also the studies which were conducted by Goldman et al on the medications price elasticity revealed that it varies from low elastic to inelastic according to medication groups so that price elasticity for the rheumatoid arthritis medications is low (-0.21) and for the cancer medications is almost none (-0.01) (Goldman et al., 2006; Goldman, Joyce, & Zheng, 2007). Also they found out that specialty drug use is largely insensitive to cost sharing. However the study of Tang et al on the estimation of governmental subsidy impact on the consumption of injection medicines in China revealed a significantly positive result (Tang et al., 2013). Based on the present study, since prescription and consumption of injection medicines in Iran is not related to price, it seems that increasing price of injection medicines -as a policy towards reducing the consumption- cannot be successful and might only bring catastrophic health expenditure for the poor.

The results of this study also revealed that the prescription of injection medicines is more related to the prescription of corticosteroids and antibiotics. It is seen that 10% increase in prescription of antibiotic and corticosteroid medications can cause 4.4 and 6.5 percent increase in prescription of injection forms of medications. So it could be concluded that policy making for reducing the use of antibiotics and corticosteroids can cause reduction in the use of injection medicines too. Unfortunately overuse of corticosteroids and antibiotics are common phenomenon in Iran. According to our study results the percentage of prescription letters containing antibiotics varies from 39 to 61 in different geographical regions. In a previous study by Soleymani et al a decreasing trend of antibiotic use in Iran was reported, this means that the number of antimicrobial drugs in prescriptions decreased 23.73% from 1998 until 2007 (Soleymani, Valadkhani, & Dinarvand, 2009). Despite the decreasing trend of the use and prescription of antibiotics in Iran, a considerable reduction is yet to be made. Also, the antibiotic resistance due to irrational and overuse of antibiotics is a major health problem (Fallahi & Maleknejad, 2007). In our study the percentage of prescription letters containing corticosteroids varies from 15 to 35 in different geographical regions. However, the results of the study by Mosleh et al on prescription of corticosteroids in Iran reveals that the consumption of corticosteroids has grown up to two times in a Ten-year period (12.68% in 1999 in comparison with 23% in 2009) (Mosleh, Darbooy, Khoshnevis Ansari, & Mohammadi, 2008). Corticosteroids Injection is a routine treatment plan and intervention to decrease pain and inflammation for many groups of diseases including rheumatoid diseases, vertebral column diseases, lung diseases, optic diseases, skin diseases and etc. (Stefanou, Marshall, Holdan, & Siddiqui, 2012). Also Anabolic Steroids abuse specially its injection form is one of the common problems among bodybuilding and powerlifting athletes. According to Nojoomi and Behravan’s study, in Iran the use of anabolic steroids among bodybuilding athletes’ is very high (26%) and in a worrying range (Nojoomi & Behravan, 2005). Corticosteroids are employed for symptomatic treatments and their widespread consumption causes 2 categories of undesirable side effects. The 1st is related to long term pharmacological doses and the 2nd is due to medication withdrawal and both could be dangerous for patients. Therefore benefits and disadvantages of their long term prescription should be considered (Mosleh, Darbooy, Khoshnevis Ansari, & Mohammadi, 2008).

The results of the present study could be a warning signal for public health systems in Iran to pay more attention to drug consumption, prescription patterns and its related factors. This subject necessarily needs much more comprehensive studies.

## 5. Conclusion

Irrational prescriptions of medicine seem to be a public health dilemma in Iran. It is said that the average number of medicines in each prescription letter and the prescription of antibiotics and injection medicines in Iran is markedly higher than the WHO standards and there are also considerable differences in prescriptions according to the different geographical regions in Iran. Thus, a comprehensive therapeutic and educational protocol is necessary in order to control and manage the consumption and prescription of medication in Iran.

In addition, the prescription of injection forms of medicine in Iran is more related to the prescription of corticosteroids and antibiotics and it is not significantly affected by price of injection medicines. Therefore increasing price of injection medicines -as a policy towards reducing consumptions- cannot be a successful policy. But reducing the use of antibiotics and corticosteroids can be a more effective policy to reduce the use of injection medicines.
